# Neurally adjusted ventilatory assist mitigates ventilator-induced diaphragm injury in rabbits

**DOI:** 10.1186/s12931-019-1265-x

**Published:** 2019-12-23

**Authors:** Tatsutoshi Shimatani, Nobuaki Shime, Tomohiko Nakamura, Shinichiro Ohshimo, Justin Hotz, Robinder G. Khemani

**Affiliations:** 1 Department of Emergency and Critical Care Medicine, Graduate School of Biomedical and Health Sciences, Hiroshima University, 1-2-3 Kasumi, Minami-ku, Hiroshima, 734-8551 Japan; 2 Division of Neonatology, Nagano Children’s Hospital, 3100 Toyoshina, Azumino City, Nagano, 399-8288 Japan; 3 Department of Anesthesiology and Critical Care Medicine, Children’s Hospital of Los Angeles, 4650 Sunset Boulevard, Los Angeles, CA 90027 United States; 4 Department of Pediatrics, University of Southern California, Keck School of Medicine, 1975 Zonal Ave, Los Angeles, CA 90033 United States

**Keywords:** Patient-ventilatory asynchrony, Ventilator-induced diaphragm dysfunction, Acute respiratory failure, Neurally adjusted ventilatory assist

## Abstract

**Background:**

Ventilator-induced diaphragmatic dysfunction is a serious complication associated with higher ICU mortality, prolonged mechanical ventilation, and unsuccessful withdrawal from mechanical ventilation. Although neurally adjusted ventilatory assist (NAVA) could be associated with lower patient-ventilator asynchrony compared with conventional ventilation, its effects on diaphragmatic dysfunction have not yet been well elucidated.

**Methods:**

Twenty Japanese white rabbits were randomly divided into four groups, (1) no ventilation, (2) controlled mechanical ventilation (CMV) with continuous neuromuscular blockade, (3) NAVA, and (4) pressure support ventilation (PSV). Ventilated rabbits had lung injury induced, and mechanical ventilation was continued for 12 h. Respiratory waveforms were continuously recorded, and the asynchronous events measured. Subsequently, the animals were euthanized, and diaphragm and lung tissue were removed, and stained with Hematoxylin-Eosin to evaluate the extent of lung injury. The myofiber cross-sectional area of the diaphragm was evaluated under the adenosine triphosphatase staining, sarcomere disruptions by electron microscopy, apoptotic cell numbers by the TUNEL method, and quantitative analysis of Caspase-3 mRNA expression by real-time polymerase chain reaction.

**Results:**

Physiological index, respiratory parameters, and histologic lung injury were not significantly different among the CMV, NAVA, and PSV. NAVA had lower asynchronous events than PSV (median [interquartile range], NAVA, 1.1 [0–2.2], PSV, 6.8 [3.8–10.0], *p* = 0.023). No differences were seen in the cross-sectional areas of myofibers between NAVA and PSV, but those of Type 1, 2A, and 2B fibers were lower in CMV compared with NAVA. The area fraction of sarcomere disruptions was lower in NAVA than PSV (NAVA vs PSV; 1.6 [1.5–2.8] vs 3.6 [2.7–4.3], *p* < 0.001). The proportion of apoptotic cells was lower in NAVA group than in PSV (NAVA vs PSV; 3.5 [2.5–6.4] vs 12.1 [8.9–18.1], *p* < 0.001). There was a tendency in the decreased expression levels of Caspase-3 mRNA in NAVA groups. Asynchrony Index was a mediator in the relationship between NAVA and sarcomere disruptions.

**Conclusions:**

Preservation of spontaneous breathing using either PSV or NAVA can preserve the cross sectional area of the diaphragm to prevent atrophy. However, NAVA may be superior to PSV in preventing sarcomere injury and apoptosis of myofibrotic cells of the diaphragm, and this effect may be mediated by patient-ventilator asynchrony.

## Background

Ventilator-induced diaphragm dysfunction (VIDD) [1–5] occurs in 30–80% of critically ill patients undergoing mechanical ventilation [6], and may be associated with prolonged mechanical ventilation and increased intensive care unit (ICU) and hospital mortality [5–7]. Major mechanisms of VIDD appear to be related to disuse atrophy from excessive respiratory support, and sarcomere injury due to eccentric contraction or load-induced injury [8–10].

Disuse atrophy has been well described to occur as soon as 12 h after controlled mechanical ventilation (CMV) in both animal and human studies [11–19]. Therefore, it is recommended that spontaneous breathing effort be preserved as a means to prevent VIDD [8], and increasingly assisted modes such as pressure support are widely used in clinical practice, although over-assistance is still possible with pressure-support ventilation.

Nevertheless, while preservation of spontaneous breathing may prevent atrophy, the risk of sarcomere injury remains, particularly if respiratory effort is high [20]. Furthermore, there is biologic plausibility that eccentric contraction of the diaphragm leading to sarcomere injury can be exacerbated by patient-ventilator asynchrony [8]. Specifically, nearly all cases of ineffective inspiratory effort occur during the expiratory phase on the ventilator. This failure to trigger mechanical breaths might result in activation of the diaphragm even while it is lengthening -i.e. eccentric contraction [21–24]. However to date, no studies have demonstrated a relationship between ventilator asynchrony and diaphragmatic injury.

Theoretically, neurally adjusted ventilatory assist (NAVA) can prevent both mechanisms of VIDD. First, the NAVA level can be adjusted to target a certain electrical activity of the diaphragm (Edi) and provide respiratory support based the patient’s respiratory effort. This can prevent both atrophy and sarcomere injury from load-induced injury. Second, numerous studies have shown improved patient-ventilator synchrony with NAVA compared to PSV [25].

We sought to determine whether NAVA resulted in less VIDD than PSV or CMV, and specifically characterize NAVA’s impact on both atrophy and sarcomere injury, and if these effects were mediated by patient-ventilator asynchrony. This was tested using an Acute Lung Injury (ALI) model in rabbits.

## Materials and methods

### Animals

Animal protocols were approved by the Institutional Animal Care and Use Committee of the Shinshu University, Nagano, Japan. Japanese white rabbits (*n* = 20; body weight, 2.4–2.9 kg) were randomly assigned to one of four groups: 1) non-ventilated animals as the control group; 2) pressure-controlled mechanical ventilation with the use of neuromuscular blockade as the CMV group; 3) NAVA group with spontaneous breathing; and 4) PSV group with spontaneous breathing. Rabbits were premedicated by intramuscular administration of midazolam (5 mg/kg) and xylazine (5 mg/kg). A 24-gage catheter was placed in the marginal ear veins and in the ear artery, the former for extracellular fluid (0.9% saline, 10 ml/kg/h) and drug administration (sodium midazolam, 3–5 mg/kg/h; xylazine, 5 mg/kg/h), and the latter for monitoring blood pressure and sampling for arterial blood gases, including pH, partial pressure of arterial carbon dioxide (PaCO_2_), and partial pressure of arterial oxygen (PaO_2_). Animals were placed in a supine position under a radiant warmer to maintain body temperature throughout the study period, and body temperature was monitored using a rectal temperature probe. For animals in the CMV, NAVA and PSV groups, endotracheal intubation was performed with 3.5 mm i.d. cuffed tube, and subsequently, experimental ALI was induced by repeated lung lavage with 25 mL/kg of normal saline in CMV, NAVA and PSV groups. ALI was considered stable when a constant PaO_2_/ fraction of inspired oxygen (F_I_O_2_) ratio of less than 300 mmHg was maintained for 30 min.

### Ventilation equipment

For NAVA and PSV groups, Edi was measured with an 8F oro-gastric catheter mounted with an array of eight electrode pairs (Maquet Critical Care, Solna, Sweden). Rabbits were ventilated at a tidal volume of 6–8 ml/kg with positive end-expiratory pressure (PEEP) of 5 cmH_2_O (Servo-i®, Maquet Critical Care, Solna, Sweden). For the CMV group, peak inspiratory pressure was adjusted to achieve the target tidal volume, and rate was adjusted to achieve a pH between 7.35 and 7.45. For the PSV group, the level of pressure support was adjusted to achieve the target tidal volume. For the NAVA group, the NAVA level was adjusted to achieve both the targeted tidal volume and a similar level of pressure support as other modes. Apnea time was set to 5 s. Flow and Edi cycle off were constant as 30 and 70% respectively. The ventilator flow and Edi triggers were set each level 5 and 0.5 μV, but no additional intervention was made to adjust synchrony. The waveforms of Airway pressure, Flow, Volume, and Edi were recorded from the ventilator using the Servo-tracker® software program (Maquet Critical Care, Solna, Sweden). The rabbits were ventilated for 12 h based on previous studies [13, 17, 19], and subsequently pentobarbital (100 mg/kg) was used to euthanize the animals for necropsy and histological evaluation.

### Physiological measurements

Heart rate, blood pressure, body temperature, percutaneous oxygen saturation (SpO_2)_, airway pressure, and Edi during mechanical ventilation were continuously measured, while arterial blood gas was measured every 6 h. Data from Servo-i® was continuously recorded by Servo Connect® (ver. 1.0.405) and analyzed offline. The number of asynchrony events (events/min) and the asynchrony index (%, asynchronous events / Edi number) were calculated for the first 5 min of each hour. The types of asynchrony were defined based on previously published definitions [26]. The degree of histological lung injury was evaluated using the lung injury score [27]. The observer who acquired physiological and respiratory data was not blinded.

### Fiber atrophy: histological assessment

Segments of the costal diaphragm and two random sections from the both lower lung lobes were removed and quick-frozen. Muscle tissue was sliced into 10 μm sections using a cryostat (HM 550, Thermo Fisher Scientific) and stained with Hematoxylin-Eosin (H&E) and with adenosine triphosphatase (ATPase). Lung tissue was stained with H&E and the method used has been reported previously [27].

### Sarcomere disruption: Electron microscopy

Muscle fibers were fixed in 2.5% glutaraldehyde, and the subsequent sample processing was performed at the Shinshu University Instrumental Analysis Support Division. Sarcomere disruption was evaluated according to the procedures described previously [10]. Micrographs of the muscle samples were taken from 15 randomly selected fields in each group using a transmission electron microscope (JEM-1400, JEOL Ltd. Japan). Sarcomere disruption was evaluated as a sign of muscle injury and defined as a zone with distinct distortion of the usual sarcomeric architecture, defined by the following six criteria: discontinuity of a group of myofibrils, A- and I-band disruption, Z-band streaming, embedded subcellular components (mitochondria or collagen), preserved adjacent sarcomere, and absence of regional sectioning artifacts (scratches, holes, or chatters). The proportion (i.e., abnormal area fraction, expressed as percentage) of sarcomere disruption was normalized to the micrographed area. The analysis of images was performed by an independent pathologist not associated with this study who was blinded to treatment group.

### Apoptosis: terminal deoxynucleotidyl transferase-mediated deoxyuridine triphosphate nick-end labeling (TUNEL) method

Frozen diaphragm muscle tissue was sectioned on a cryostat and stained using an In situ Apoptosis Detection Kit (MK 500, Takara bio Inc., Japan) according to the manufacturer’s instructions. Methyl green was used for counterstaining. Apoptotic cell ratio was calculated as the ratio of positive cell nucleus to total cell nucleus number in randomly selected fields.

### Muscle protein degeneration: measurement of messenger ribonucleic acid (mRNA) expression by quantitative realtime polymerase chain reaction (PCR)

Caspase-3 is a protease that plays an important role in the development of apoptosis and is involved in muscle protein degeneration during muscle fatigue. Muscle tissue samples were stored in ribonucleic acid (RNA) Save (Biological Industries Ltd. Israel), homogenized with Biomasher 2 (Nippi inc. Japan), and total RNA extracted with EZ-RNA Total RNA Isolation Kit (Biological Industries Ltd. Israel). RNA purity was measured with an Agilent 2100 Bioanalyzer System (Agilent Technologies, Inc. Santa Clara, Calif., USA) and complementary deoxyribonucleic acid (cDNA) was prepared using SuperScript™ IV VILO™ Master Mix with ezDNase™ (Thermo Fisher Scientific Inc., Waltham, Mass., USA). After previously verifying amplification efficiency, expression level of Caspase-3 was measured by the ΔCt method with glyceraldehyde-3-phosphate dehydrogenase (GAPDH) as endogenous control.

Primers used are as follows:

GAPDH:5′-CGCCTGGAGAAAGCTGCTA-3′, 5′ -ACGACCTGGTCCTCGGTGTA-3′.

Caspase 3 [28]: 5′-GCTGGACAGTGGCATCGAGA-3′, 5′-TCCGAATTTCGCCAGGAATAGTAA-3′.

Realtime PCR was performed using the PowerUP SYBR Green PCR Master Mix (Thermo Fisher Scientific Inc., Waltham, MA, USA) on a StepOnePlus™ system (Thermo Fisher Scientific Inc., Waltham, MA, USA).

### Statistical analysis

Image analysis was performed by an independent pathologist not associated with this study who evaluated the tissue using Image J (National Institutes of Health, Bethesda, Maryland, USA). Normality of the data was tested by the Shapiro–Wilk test, the Mann-Whitney’s *U* test was used for comparing 2 groups, and the Kruskal–Wallis test was used for comparing 3 or more groups. Post-hoc comparisons were made using the Bonferroni method. Correlation was tested by the Spearman’s rank correlation. Multiple linear regression was used for mediation analysis, evaluating the change in coefficient of treatment group with the introduction of a potential mediator variable. R version 3.5.0 (2018 The R Foundation for Statistical Computing Platform) was used for statistical analysis and all tests were considered to be statistically significant when *p*-value was < 0.05.

## Results

### Physiological measurements

Data on vital signs, respiratory parameters, and lung injury score are summarized in Table 1. With respect to vital signs, systolic blood pressure was slightly higher in the NAVA group at the beginning of the experiment with no significant difference in the subsequent values. Peak inspiratory pressure was slightly higher in the CMV compared to NAVA and PSV groups, but this was not significant in multiple-comparisons post-hoc analysis. PaO_2_/F_I_O_2_ and post-necropsy histologic lung injury score were not significantly different between groups.

**Table 1:** Physiological measurements in the control, CMV, NAVA, and PSV groups

	Time (hours)	Control	CMV	NAVA	PSV	*p* value^a^
Body weight (kg)	NA	2.6 [2.6–2.7]	2.7 [2.5–2.7]	2.8 [2.7–2.8]	2.8 [2.5–2.8]	0.21
Heart rate (bpm)	0	138 [130–150]	142 [126–157]	116 [110–124]	127 [126–150]	0.36
	6	NA	163 [140–200]	138 [132–144]	148 [145–158]	0.43
	12	NA	180 [156–181]	134 [130–160]	144 [135–146]	0.37
Systolic blood pressure (mmHg)	0	82 [80–90]	85 [80–90]	102 [95–105]	84 [77–90]	0.035
	6	NA	85 [80–88]	79 [79–100]	80 [75–86]	0.91
	12	NA	87 [80–91]	86 [81–87]	77 [70–88]	0.69
Diastolic blood pressure (mmHg)	0	58 [58–60]	70 [65–70]	75 [72–82]	68 [56–72]	0.055
	6	NA	60 [54–68]	50 [50–63]	58 [57–61]	0.93
	12	NA	62 [60–71]	59 [54–60]	56 [45–59]	0.29
Body temperature (°C)	0	38.8 [38.2–38.9]	38.1 [37.4–38.2]	38.5 [38.5–38.9]	38.4 [38.4–38.5]	0.19
	6	NA	38.7 [38.1–39.0]	38.5 [38.5–38.8]	39.2 [38.7–39.7]	0.39
	12	NA	39.0 [38.3–39.2]	38.6 [38.2–39.3]	39.4 [38.9–39.5]	0.51
PaO_2_/F_I_O_2_	0	NA	264 [244–275]	251 [232–293]	276 [275–285]	0.74
	6	NA	257 [209–258]	271 [257–281]	259 [257–261]	0.37
	12	NA	255 [220–267]	283 [268–303]	253 [248–259]	0.11
PaCO_2_ (mmHg)	0	NA	42 [39–50]	37 [36–41]	39 [37–45]	0.48
	6	NA	44 [41–48]	39 [37–43]	40 [38–46]	0.36
	12	NA	43 [43–45]	41 [38–41]	45 [35–46]	0.54
Respiratory rate (rpm)	NA	48 [38–60]	40 [40–55]	42 [35–51]	45 [39–59]	0.86
Peak inspiratory pressure (cmH_2_O)	NA	NA	14.0 [12.9–15.7]	12.2 [10.8–14.1]	12.6 [11.0–14.5]	0.045
Tidal volume (mL/kg of body weight)	NA	NA	7.6 [7.1–8.4]	6.8 [6.2–7.8]	7.1 [6.1–8.4]	0.10
Edi peak (μV)	NA	NA	NA	4.7 [2.2–7.4]	4.9 [2.8–8.7]	0.35
Edi min (μV)	NA	NA	NA	1.6 [0.6–3.3]	1.8 [0.9–3.2]	0.55
Pressure support (cmH_2_O)	NA	NA	NA	NA	7 [5–8]	NA
NAVA level (cmH_2_O/μV)	NA	NA	NA	1.6 [1.0–1.9]	NA	NA
Lung injury score	NA	NA	3.0 [2.8–3.3]	2.5 [1.8–3.8]	3.0 [2.0–4.0]	0.97

values are expressed as median [interquartile range]

Respiratory rate, peak inspiratory pressure, tidal volume, edi peak, edi min, pressure support and NAVA level represent the median over 12 h for all of the observed parameters.”

^a^ Kruskal-Wallis test

*CMV* control mechanical ventilation, *NAVA* neurally adjusted ventilatory assist, *PSV* pressure support ventilation; bpm, beats per minutes, *NA* not available, *PaO*_*2*_ partial pressure of arterial oxygen, *F*_*I*_*O*_*2*_ fraction of inspired oxygen, *PaCO*_*2*_ partial pressure of arterial carbon dioxide; Edi, electrical activity of diaphragm

### Asynchrony

For the spontaneously breathing groups (NAVA and PSV), ineffective efforts were less common in the NAVA group compared with the PSV group (median [interquartile range], NAVA group vs PSV group; 0 events [0–0.3] vs 2.0 events [1.0–3.5], *p* = 0.003) (Table 2). There were no cycle or flow asynchronies noticed. Asynchrony index, which corresponds to the total number of asynchrony events/number of Edi signals × 100, was also significantly lower in the NAVA group (NAVA group vs PSV group; 1.1 [0–2.2] vs 6.8 [3.8–10.0], *p* = 0.023) (Table 2).

**Table 2:** Asynchrony with mechanical ventilation in NAVA and PSV

	NAVA	PSV	*p* value^a^
Ineffective inspiratory efforts (events/min)	0 [0–0.3]	2.0 [1.0–3.5]	0.003
Auto-triggering (events/min)	0 [0–1.0]	0 [0–1.0]	0.057
Double triggering (events/min)	0 [0–0.3]	0 [0–0]	0.68
Asynchrony index (%)	1.1 [0–2.2]	6.8 [3.8–10.0]	0.023

values are expressed as median [interquartile range]

^a^ Mann–Whitney’s *U* test

*NAVA* neurally adjusted ventilatory assist, *PSV* pressure support ventilation

### Atrophy

**Fig. 1:**
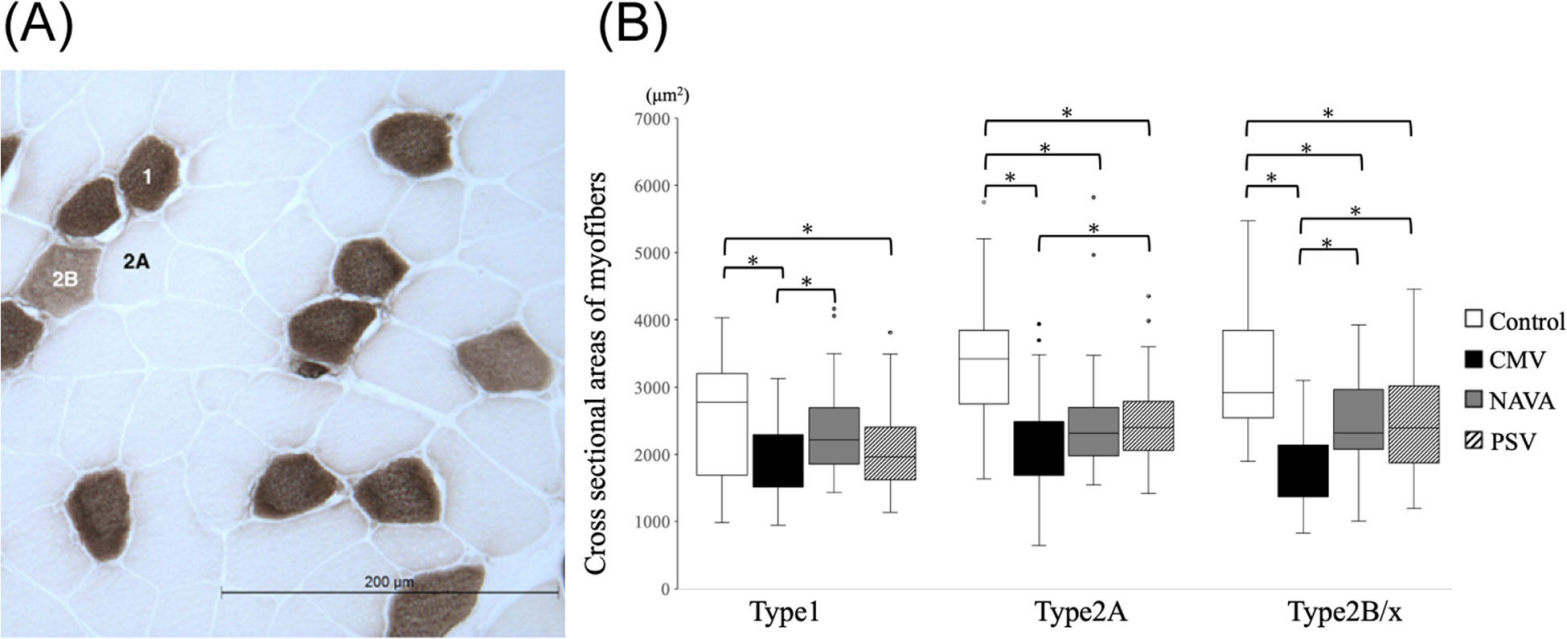
Comparison of cross-sectional area of myofibers among the four groups. Compared to the other three groups, in the CMV group cross-sectional area was lower in all fiber Types (1, 2A, 2B/x). There was no significant difference between the NAVA and the PSV group (adenosine triphosphatase staining **a**). (white column: Control, black column: CMV, grey column: NAVA, stripe column: PSV **b**) *: *P* < 0.05, adjusted for the Bonferroni method

In general, fiber cross-sectional area in the diaphragm (a measure of atrophy) was significantly lower in the ventilated rabbits (all groups) compared to control rabbits. CMV resulted in the most atrophy of all fiber types relative to control rabbits, and both NAVA and pressure support appear to have more preservation of cross sectional area in the diaphragm compared to the CMV group. There were no significant differences in fiber cross sectional area between the NAVA and the PSV groups (Fig. 1).

### Sarcomere disruption

**Fig. 2:**
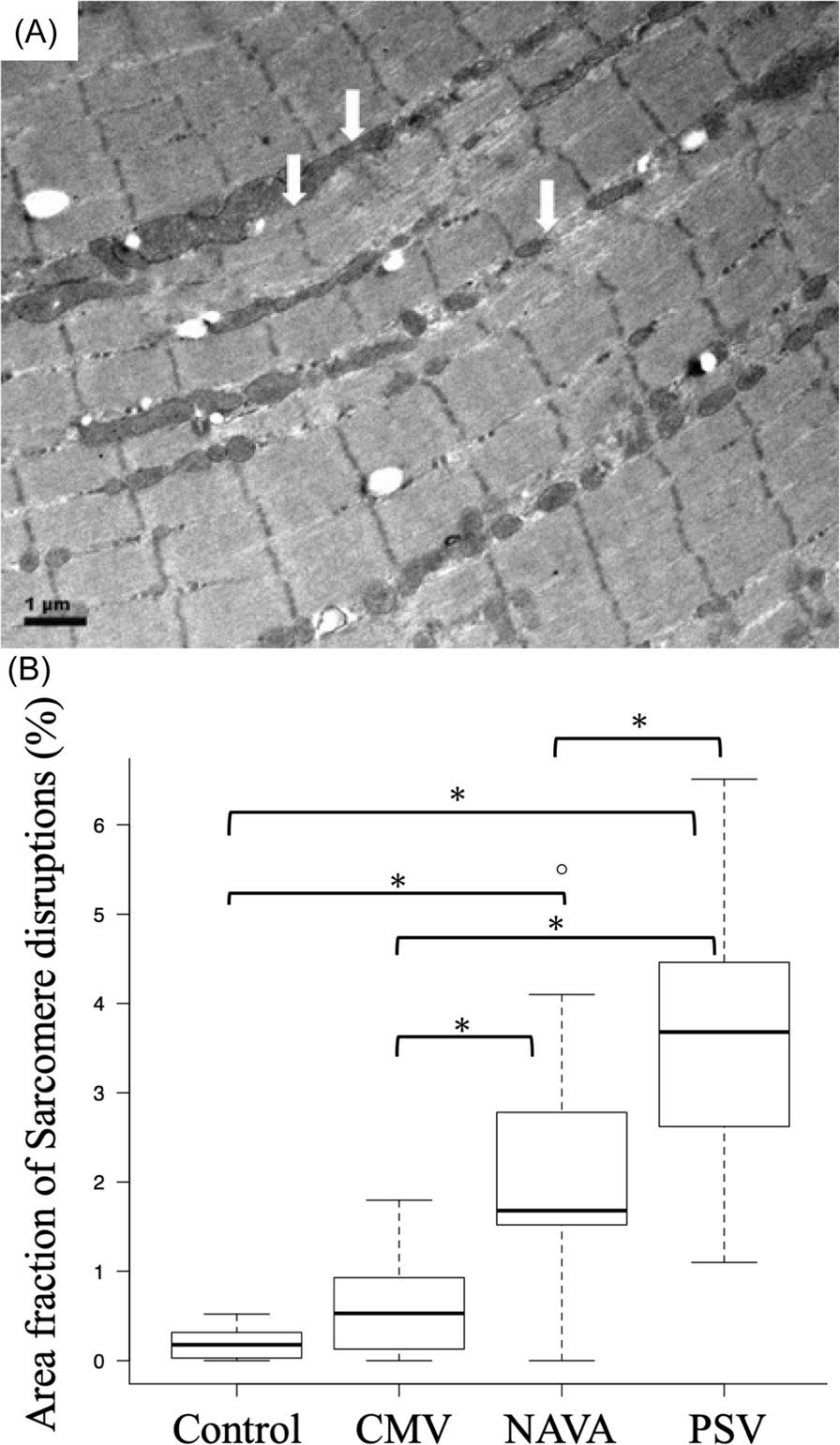
Comparison of area fraction of sarcomere disruptions among the four groups. Z-streaming (white arrows) indicate sarcomere disruptions in the longitudinal profile of the muscle fibers **a**. The area fraction of sarcomere disruptions was significantly higher in the PSV group compared with the NAVA group. In the CMV group, sarcomere disruptions were lower than those seen in the NAVA and PSV groups **b**. *: *P* < 0.05, adjusted for the Bonferroni method

The area fraction of sarcomere disruptions was similar between control rabbits and those on CMV. Both NAVA and PSV had more sarcomere disruptions than CMV or control rabbits, with the PSV group having the highest degree of sarcomere disruptions (area fraction of sarcomere disruptions, control vs CMV vs NAVA vs PSV: 0.1 [0.02–0.3] vs 0.5 [0.1–0.8] vs 1.6 [1.5–2.8] vs 3.6 [2.7–4.3], *p* < 0.001) (Fig. 2).

### Apoptosis

**Fig. 3:**
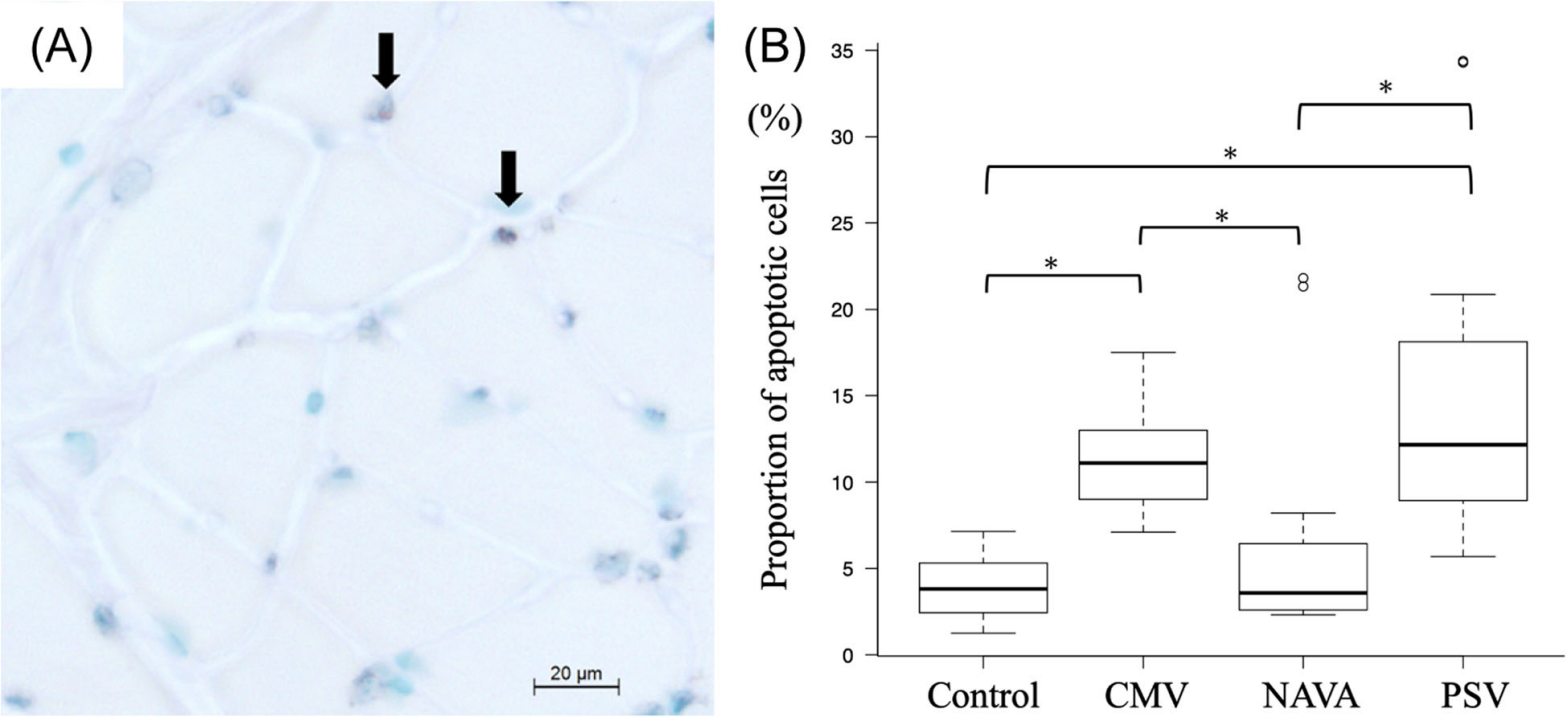
Apoptotic cell ratio among the four groups measured by the TUNEL method. Apoptotic cell ratio stands for the number of positive cells/total number of cells. Black arrows indicate positive cells **a**. NAVA had a significantly lower apoptotic cell ratio compared with CMV or PSV **b**. (Methyl green was used as a counterstain) *: *P* < 0.05, adjusted for the Bonferroni method

The proportion of apoptotic cells was similar between control and NAVA groups, but significantly higher in both CMV and PSV (control vs CMV vs NAVA vs PSV: 3.8 [2.5–4.9] vs 11.1 [9.0–12.9] vs 3.5 [2.5–6.4] vs 12.1 [8.9–18.1], *p* < 0.001) (Fig. 3).

### Muscle protein degradation

There was a tendency in the decreased expression levels of Caspase-3 mRNA in NAVA groups (CMV vs NAVA vs PSV, compared with the baseline control group: 15.0 [5.7–22.6] vs 11.1 [7.96–17.3] vs 27.6 [18.2–52.6], *p* = 0.065).

### Correlation and mediation analysis

**Fig. 4:**
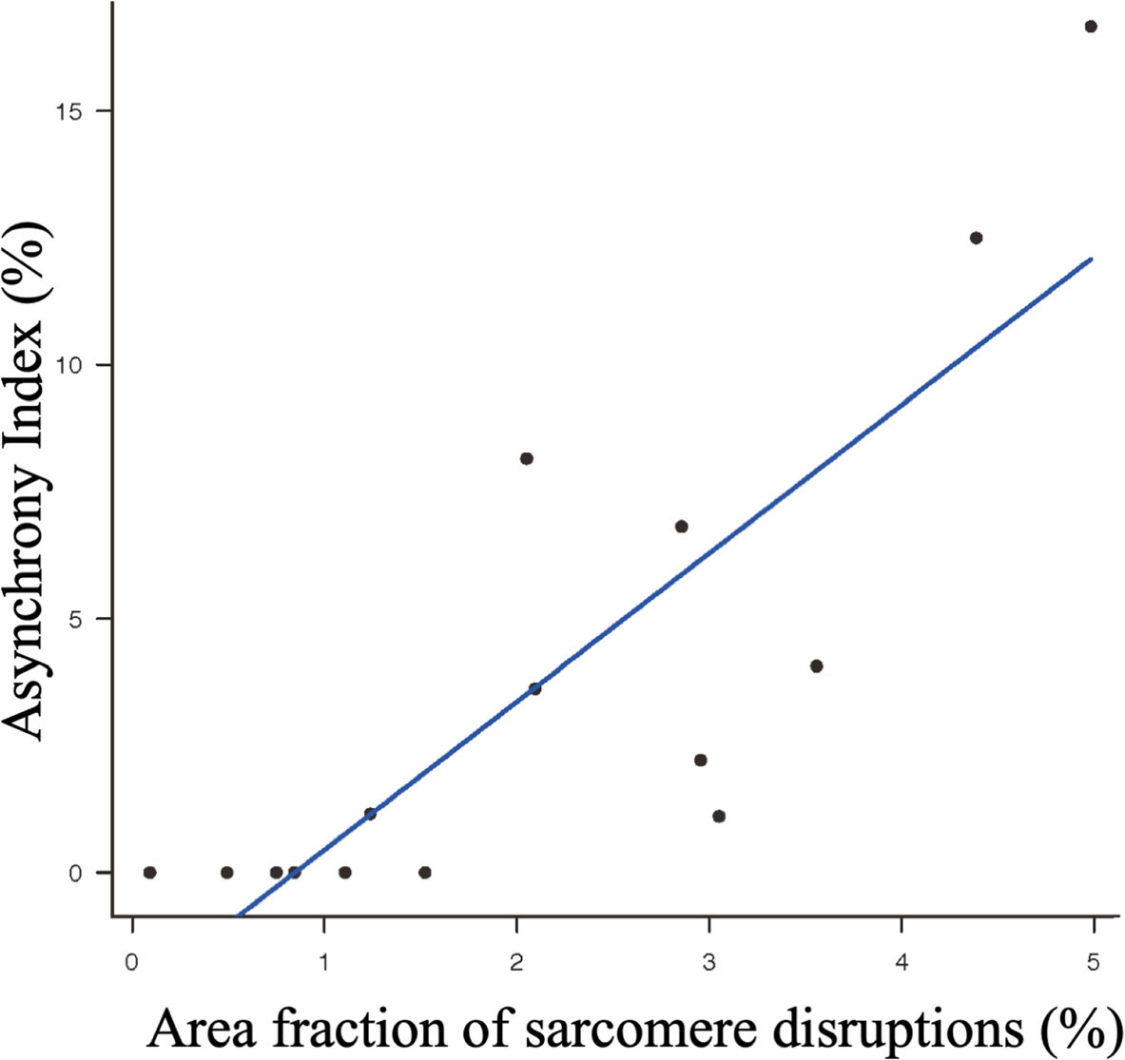
Correlation between Asynchrony Index and sarcomere disruptions. AI is strongly correlated with sarcomere disruptions (AI and area fraction of sarcomere disruptions; coefficient 0.834, *p* = 0.0001)

There was a strong correlation between the asynchrony index (AI) and sarcomere injury (AI and area fraction of sarcomere disruptions; coefficient 0.834, *p* = 0.0001) (Fig. 4). When we built a multivariable model with group variety (NAVA vs PSV) and AI, on the outcome of sarcomere disruptions, we found that the effect that protective effect NAVA had on sarcomere disruptions appears to be heavily mediated by Asynchrony Index, as NAVA was no longer significant in the multivariable model after controlling for AI (Table 3).

**Table 3:** Mediation analysis with Multiple linear regression

Response variable	Factors	B	SE	t	*p* value	95% CI
Sarcomere disruptions	NAVA	−0.176	0.651	−0.270	0.789	−1.502 - 1.151
	AI	0.196	0.061	3.200	0.003	0.071–0.321
Sarcomere disruptions	NAVA	1.051	0.486	2.161	0.038	0.060–2.042
	Edi peak	0.227	0.106	2.146	0.039	0.012–0.443

*NAVA* Group variety NAVA vs PSV, *AI* Asynchrony Index, *B* regression coefficient, *SE* Standard Error, *CI* Confidence interval

There was also a positive correlation between Edi and sarcomere injury (Edi and area fraction of sarcomere disruptions; coefficient 0.622, *p* = 0.013). However, both NAVA and Edi retained a significant association with sarcomere disruption in a multivariable model, implying that Edi does not mediate the protective relationship between NAVA and sarcomere disruption (Table 3). In fact, when developing a single multivariable model, only Asynchrony Index retained an independent association with sarcomere disruption (Table 4).

**Table 4:** Multivariable model

Response variable	Factors	B	SE	t	*p* value	95% CI
Sarcomere disruptions	NAVA	−0.110	0.700	− 0.158	0.876	−1.537 - 1.317
	AI	0.181	0.082	2.201	0.035	0.013–0.348
	Edi peak	0.037	0.132	0.283	0.779	−0.232 - 0.307

*NAVA* Group variety NAVA vs PSV, *AI* Asynchrony Index, *B* regression coefficient, *SE* Standard Error, *CI* Confidence interval

There was no significant correlation among CSA, AI, and Edi, except between AI and CSA 2B/x (Table 5).

**Table 5:** Correlation coefficient among CSA, AI, and Edi

	CSA 1	CSA 2A	CSA 2B/x
AI	−0.247, *p* = 0.375	0.098, *p* = 0.729	0.601, *p* = 0.018^a^
Edi peak	0.124, *p* = 0.661	−0.004, *p* = 0.99	0.444, *p* = 0.098

*AI* Asynchrony Index, *CSA* fiber cross-sectional area in the diaphragm

^a^Spearman’s rank correlation

## Discussion

We have shown that preservation of spontaneous breathing prevents diaphragm atrophy and NAVA leads to lower sarcomere injury in the diaphragm compared with PSV. Based on the fact that Edi levels were similar between PSV and NAVA groups, and that the benefit of NAVA on lower sarcomere injury appears to be mediated by the Asynchrony Index, we believe NAVA may have lessened sarcomere injury due to prevention of eccentric contraction. To the best of our knowledge, this is the first study that describes how sarcomere injury can be mitigated by improving synchrony.

Mechanisms underlying VIDD include atrophy due to excessive breathing assistance and/or sarcomere injury. We found that all forms of ventilation are associated with reduction in the cross-sectional area of muscle fibers compared to non-ventilated control animals, reinforcing previous findings that mechanical ventilation is associated with diaphragm atrophy. Furthermore, both NAVA and PSV were associated with less atrophy than CMV, likely because spontaneous breathing was preserved. However, we saw no difference in the degree of atrophy between the NAVA and PSV groups. This is likely because Edi levels were similar between NAVA and PSV groups, indicating similar degrees of respiratory effort. It is possible that selecting a different level of pressure support, or a different Edi level could have prevented this atrophy completely. This should be a focus of future research.

In contrast, area fraction of sarcomere disruptions, along with proportion of apoptotic cells, were lower in the NAVA group than in the PSV group. In our study, we found that ineffective inspiratory efforts, which can produce fierce eccentric contraction, were significantly reduced in NAVA groups. We saw similar Edi levels in both NAVA and PSV groups, and Edi did not appear to mediate the relationship between NAVA and less sarcomere disruption in our multivariable model. In contrast, the degree of asynchrony did appear to mediate this relationship, which suggests a role for asynchrony in VIDD. Furthermore, previous studies have demonstrated that nuclear damage and apoptosis could occur in sarcomere injury [10, 26, 29]. As such, our finding that the proportion of apoptotic cells was decreased in the NAVA group compared with the PSV group seemed consistent with the previous studies [30]. Interestingly the rates of apoptosis in the CMV group were similar to the PSV group, and higher than the NAVA group. This is probably because of apoptosis due to atrophy, as CMV showed significantly lower cross-sectional area of myofibers for each fiber type.

There was a tendency of higher expression in the PSV group than in the NAVA group. Caspase-3 is a protease that plays an important role in the development of apoptosis and is involved in muscle protein degeneration during muscle fatigue. During mechanical ventilation, Caspase-3 is activated in the diaphragm, and this has been demonstrated both in animal models and in humans [17, 18]. Therefore, it can be an indicator of load-induced injury of the diaphragm.

Previous animal studies support that NAVA is associated with fewer asynchronous events and lower diaphragmic load compared to PSV, using a rabbit lung injury model. PSV had a greater trigger delay than NAVA (PSV, 90–228 ms; NAVA, 76–96 ms; *p* < 0.05), implying that the transdiaphragmatic pressure-time product was higher in PSV [31]. Campoccia, Jalde et al. evaluated the effects of NAVA and PSV on ventilation efficiency, respiration pattern, and dead space in healthy rats, and reported that NAVA reduced dead space, and increased minute ventilation and ventilation efficiency [32]. Both these studies have shown that NAVA was superior to PSV in terms of neuro-ventilatory coupling. However, to date, as no animal experiments have evaluated the effects of NAVA and PSV on VIDD, our study provides new mechanistic insights.

Asynchrony is a potentially serious event that may be overlooked even by an experienced intensive care physician [33] and many studies have compared NAVA and PSV with respect to clinical parameters such as asynchrony. Demoule et al. performed a multicenter randomized controlled trial (RCT) on comparing NAVA and PSV in the early phase of weaning from mechanical ventilation, and have reported a decrease in asynchrony index with NAVA [34] (NAVA, 14.7; PSV, 26.7%; *p* < 0.001). Recently, a meta-analysis [35] on examining whether proportional modes improved patient–ventilator interaction has revealed that NAVA reduced a risk of asynchrony by 0.20 (95% confidence interval, 0.05–0.92). It is essential to identify ventilator settings most suited to the patient based on clinical findings and graphics. We also suggest that the reduction of asynchrony by using NAVA was important for adequate patient-ventilator interaction. Di Mussi et al. compared in a RCT that the diaphragm function in NAVA and PSV in terms of neuro-ventilatory efficiency (tidal volume/Edi) and neuro-mechanical efficiency (pressure generated against the occluded airways/Edi) [36] and reported an improvement in diaphragm function at 24 and 48 h after starting NAVA. Our histological findings support these results.

There are several limitations in this study. We did not evaluate physiological parameters including muscle contraction force, so it is unclear if this degree of sarcomere injury will correlate to clinical outcomes. Future studies should aim to evaluate the clinical impact of sarcomere injury. We limited mechanical ventilation to 12 h, supported by previous investigations. It is likely that the magnitude of these effects will be more prominent during longer periods of mechanical ventilation, although this is speculative. We did not make adjustments to PEEP level or flow cycle off percentage in an attempt to improve synchrony on PS. It is possible that making such adjustments could have had a similar effect as NAVA on reducing AI, and could have prevented sarcomere disruption. Finally, group sizes were small and in one species, which limits the generalizability of our findings. Nevertheless, we believe the important finding is that asynchrony, and in particular ineffective triggering is associated with sarcomere disruption. Methods to reduce this type of asynchrony should therefore be considered in clinical practice, NAVA being one of these methods.

## Conclusions

We have demonstrated that preservation of spontaneous breathing prevents diaphragm atrophy and NAVA may be superior to PSV in preventing sarcomere injury and apoptosis of myofibrotic cells of the diaphragm, and that this effect may be mediated by patient-ventilator asynchrony mostly related to ineffective efforts, and not the degree of inspiratory load. These results suggest potential unfavorable effects of patient-ventilator asynchrony on diaphragm function.

## Data Availability

The datasets used and/or analysed during the current study are available from the corresponding author on reasonable request.

## Abbreviations

AI: Asynchrony index
ALI: Acute lung injury
ATPase: Adenosine triphosphatase
cDNA: Complementary deoxyribonucleic acid
CMV: Control mechanical ventilation
Edi: Electrical activity of the diaphragm
F_I_O_2_: Fraction of inspired oxygen
GAPDH: Glyceraldehyde-3-phosphate dehydrogenase
H&E: Hematoxylin-Eosin
ICU: Intensive care unit
mRNA: Messenger ribonucleic acid
NAVA: Neurally adjusted ventilatory assist
PaCO_2_: Partial pressure of arterial carbon dioxide
PaO_2_: Partial pressure of arterial oxygen
PCR: Polymerase chain reaction
PEEP: Positive end-expiratory pressure
PSV: Pressure support ventilation
RCT: Randomized controlled trial
RNA: Ribonucleic acid
SpO_2_: Percutaneous oxygen saturation
TUNEL: Terminal deoxynucleotidyl transferase-mediated deoxyuridine triphosphate nick-end labeling
VIDD: Ventilator-induced diaphragm dysfunction
